# A Bayesian network approach to evaluating footwear evidence

**DOI:** 10.1016/j.fsisyn.2026.100673

**Published:** 2026-05-11

**Authors:** Danyela Kellett, David Lagnado, Ruth Morgan, Sherry Nakhaeizadeh

**Affiliations:** a Department of Security and Crime Science, University College London, 35 Tavistock Square, London, WC1H 9EZ, UK; b Scientific Support Department, Lancashire Constabulary, Saunders Lane, Hutton, PR4 5SB, UK; c UCL Centre for the Forensic Sciences, University College London, 35 Tavistock Square, London, WC1H 9EZ, UK; d UCL Arista Institute, Faculty of Engineering Sciences and Faculty of Arts and Humanities, Torrington Place, London, WC1H 7JE, UK; e Department of Experimental Psychology, UCL, 26 Bedford Way, London, WC1H 0AP, UK

**Keywords:** Footwear, Forensic, Bayesian network, Interpretation, Evaluation, Likelihood ratio

## Abstract

The interpretation, evaluation and communication of forensic footwear findings has had an impact on its use and the perception of its value, although this is also an endemic issue in the wider forensic science discipline. Tools for presenting forensic evidential findings in a clear, robust and transparent manner, such as Bayesian Networks, have been proposed, but their use in operational forensic science in the United Kingdom is limited due to the perceived complexity with building and populating such models. This paper aims to present an overview of the current method of footwear analysis and interpretation in England and Wales, which can lead to challenges in forensic evaluation, provide examples of where these issues are encountered operationally, and suggest a Bayesian Network model that could address these problems, both in forensic footwear examination and also in broader forensic science practice. A number of models are presented and populated with data from operational databases and with qualitative information provided by an operational forensic footwear expert currently working in the UK. The R v T judgment is used as an example of how such a tool could help to deliver more transparency when communicating forensic evidence and outcomes to the wider Criminal Justice System. A sensitivity analysis of data from different sources is shown, to demonstrate the potential range of values that could be reached for the likelihood ratio and a suggestion of how this could be presented as upper and lower bounds is made.

## 1 Introduction

Footwear marks are one of the most commonly encountered types of evidence at crime scenes [1,2]. Whilst an offender can wear gloves to avoid leaving fingerprints and may not leave DNA, it is difficult not to leave footwear marks. Moreover, because these marks are often latent, the offender may be unaware of their presence and therefore make no attempt to remove them [3,4]. Nevertheless, dependent on the priorities and policies of a law enforcement agency, footwear evidence may not be routinely recovered by Crime Scene Investigators [5]. The use of footwear evidence has been affected by various factors, including changing police priorities, where focus has moved away from the detection of acquisitive crime to public protection and prevention, in addition to austerity measures that have led to cuts in forensic budgets [6]. Issues with how footwear evidence is reported and communicated has also had an impact on the perception of its value, in part due to a notorious appeal court ruling in 2010 [7], but this is also an issue endemic to forensic science more widely [8,9].

A widely discussed potential solution to the issue of evaluating forensic evidence and communicating the findings is the use of Bayesian Networks (BN) [[10], [11], [12], [13]]. Bayesian Networks provide a graphical representation of the probabilistic relationships between multiple variables, including the dependencies between them, and allow the user to update their hypotheses based on new evidence [11,14,15].

**Fig. 1:**
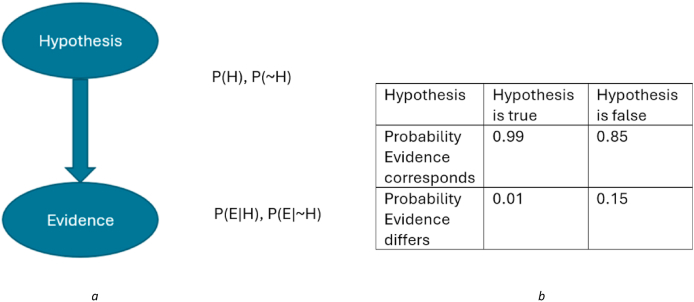
Simple two-node Bayesian Network. Fig. 1a is a graphical representation, where the upper (parent) node represents the hypothesis and the lower (child) node represents the evidence; the arrow represents the probabilistic relationship between them, in this case that the evidence node depends on the hypothesis node. Here, the hypothesis is binary: either true or false. The evidence is also binary; with a conditional probability table (CPT), Fig. 1b, which shows the probability of the different outcomes for the evidence given different states of the hypothesis.

Bayesian Networks are made up of two components, as shown in Fig. 1: (i) a directed acyclic graph that captures the relations between variables, using nodes to represent the variables and arrows to represent the dependencies between variables, and (ii) a set of probability tables for each node that quantify how each variable depends on its immediate causes [14]. This enables a Bayesian Network to be used to answer queries about the probability of a hypothesis given the observed evidence and also to predict what evidence would be expected in a particular scenario [16]. The Conditional Probability Table (CPT) can be linked to values from databases or populated using the practitioner's judgment on the probability of different outcomes [17].

Bayesian models for other forensic science fields, including biology [18] and soil comparison [19], and for combining evidence to support activity level evaluations have been proposed [[20], [21], [22], [23]]. However, this research has not yet been successful in bringing this capability into operational forensic evaluation and crime reconstruction in England and Wales. Furthermore, although there is some research published in relation to the specific issues relating to forensic footwear interpretation [[24], [25], [26], [27], [28]], none propose the structure of a Bayesian Network to support footwear evaluation.

Therefore, this paper first presents an overview of the key nuances of footwear analysis which can pose challenges in forensic evaluation and crime reconstruction endeavours, second, offers an example of where these issues arise in casework, and third presents a Bayesian Network model that could address these problems and also be applicable in forensic science practice.

## 2 Forensic footwear examination and interpretation

### 2.1 Background to interpretation of footwear evidence

The interpretation of findings in forensic science requires reasoning in the face of uncertainty, as both the forensic scientist and the court are striving to determine what happened at a crime scene with incomplete and often unreliable information [29]. The standard approach to evaluating evidence under uncertainty is given by the Bayesian framework [21,30]. The approach was introduced into operational use in England and Wales by the Forensic Science Service (FSS), as part of the development of the concept of case assessment and interpretation to provide a balanced, logical and robust way to evaluate the findings in forensic casework [31]. This approach was set against a backdrop of the commoditisation of forensic science evidence and the development of DNA evidence, with a need to communicate the significance of “matching” profiles [31,32]. The initial approach was extended to other evidence types, including footwear [25]. Reporting Officers (ROs) at the FSS were trained to use the LR approach, to evaluate their findings and produce a conclusion which articulated the relevant probative value of the evidence as a strength of support for one of a pair of competing hypotheses.

Other methods of presenting or evaluating forensic science evidence in general, and footwear evidence specifically, are used, both in the UK and worldwide [[33], [34], [35], [36]], including an expert's individual opinion of the significance of the findings [37], frequently used in the evaluation of fingerprint evidence; the US method for footwear evidence whereby the presence and extent of class and/or identifying characteristics is considered [36]; and the “sufficient agreement” theory, sometimes used in tool mark comparisons [38]. Moreover, different methods of communicating the strength of the evidence, following the use of LR or other methods to evaluate are in use, including the verbal scale of support, a statement of the relative probability of the findings or a numerical scale [33,39].

For footwear evidence, the findings are usually considered at “source” level; this is the first of three levels of propositions, known as the hierarchy of propositions [22,40] and relates to the likelihood that the evidence recovered from a crime scene originated from the suspect's footwear or alternatively, from the footwear of an unknown person [41]. This is usually expressed as the prosecution proposition being “the mark was made by the footwear of X” and the alternative proposition being “the mark was made by some other, unknown footwear” [33].

Footwear examinations comprise a comparison of the mark or marks from the scene with footwear recovered from a suspect [42,43]. Test impressions are made of the footwear in such a way as to provide a comparable mark to the crime scene [43,44]. The crime scene mark and test impression are then compared in terms of several properties focusing on four main areas of characteristics of the footwear: *pattern type*, *size & configuration, wear,* and *damage* [45].

When a comparison is made between a mark and the test impressions produced from the footwear of a suspect, the examiner will evaluate whether the test impression corresponds with the mark in each of these four categories and, if so, to what extent [43,46,47]. If differences are noted, there may be an explanation for this [43,44]. For example, a mark may appear to have been made by a larger shoe than the suspect's footwear because of movement in the mark or because the mark has been made with a wet shoe which has caused the mark to expand. Furthermore, if there is a time delay from when the mark was deposited at the crime scene to the recovery of the footwear, it is feasible that the footwear may have become more worn in the intervening period [44].

If the footwear examiner identifies inexplicable differences in the characteristics observed at any stage of the comparison, the footwear will be excluded as having made the mark and the examination will conclude [42]. If the footwear cannot be excluded as having made the mark, either because there are no significant differences or because those differences that are observed can be explained, it is necessary to evaluate the significance of the correspondence [33]. Each property is taken individually and the examiner will endeavour to determine a value for the LR for each [24].

These individual LRs are then multiplied to produce an overall LR for the footwear comparison [24,48]. This can be expressed as:

(1) LR=P×S×W×D

Where *LR* = overall LR for the footwear comparison.

*P* = LR for the pattern type.

*S* = LR for the size & configuration.

*W* = LR for the degree & distribution of wear.

*D* = LR for damage.

According to probability theory, calculating a LR for each of these properties and then multiplying them to produce the overall LR for the comparison requires that each of the properties are conditionally independent of each other [19]. This may not be the case for the properties of an item of footwear; this issue is discussed in further detail later in this paper.

The LR for each property is evaluated using various resources including the examiner's experience, knowledge and judgement [47], reference data from other cases or footwear and data on the frequency of occurrence of particular features or properties of the footwear in a relevant population [49]. Champod et al. (2004) [49] describe three different databases that could be considered to comprise a relevant population. The first is an offender-related database, which would include information relating to individuals arrested during criminal investigations. The second is an innocent suspects database, comprising information relating to people who have not been arrested but have come to notice during the investigation. The third is a crime-related database, which would consist of information relating to evidence recovered from crime scenes similar to that being considered. For footwear, Champod et al. (2004) [49] suggest that the offender-related database is the most appropriate.

Such a database can be collated by retaining footwear prints from suspects in cases submitted for comparison, or by collecting footwear prints from detainees in custody [50]. The latter option also has the benefit of providing intelligence information [51]. It has been argued that data from a manufacturer or sales database may be more appropriate, but an offender-related database is more relevant, as it considers the types of footwear worn by those who have committed crimes, or have been suspected of committing crimes [25,49]. There may be some property of footwear that makes them more suitable for those committing crimes, such as it allows freedom of movement for the wearer, or it may be that some types of footwear are more popular and fashionable within the demographic that overlaps with that of offenders [52]. Such footwear would be expected to be encountered more frequently in a forensic database than in a general sales database, giving a higher probability of observing this pattern if the mark was made by an unknown shoe and therefore a more conservative value for the LR. A database from sales or manufacturer information would also include shoes which are rarely if ever encountered at crime scenes, such as high-heels, slippers, children's shoes and so on, which would provide data irrelevant to the population being considered [50].

### 2.2 Pattern type

*Pattern type* refers to the manufacturer's design on the undersole of the shoe [53], of which there are currently over 58,000 different examples on the National Footwear Reference Collection (NFRC) [54]. The numerator in the LR for pattern type must, by definition, be close to 1; if the footwear mark was indeed made by the considered footwear, then the mark must be of the same pattern type as the footwear [50]. The denominator is the probability of the findings if the mark were made by some other unknown shoe, which equates to the probability of the findings if the suspect's shoe did not make the mark but is the same pattern type by chance [50]. This value is usually reached by interrogating a relevant database to determine the relative frequency of the pattern. For example, if the database contains 10,000 entries of which 500 are of the pattern of interest, then the probability of a chance pattern match is 500/10000 [24]. The LR for the pattern only is the probability of observing matching patterns given that the mark was made by the suspect's shoe, which is 1, divided by the probability of observing matching patterns given that the mark was made by some other shoe, which is 500/10000. This reduces to 20.

$$LR=\frac{1}{500/10000}$$

$$LR=\frac{10000}{500}$$

LR=20

### 2.3 Size & configuration

**Fig. 2:**
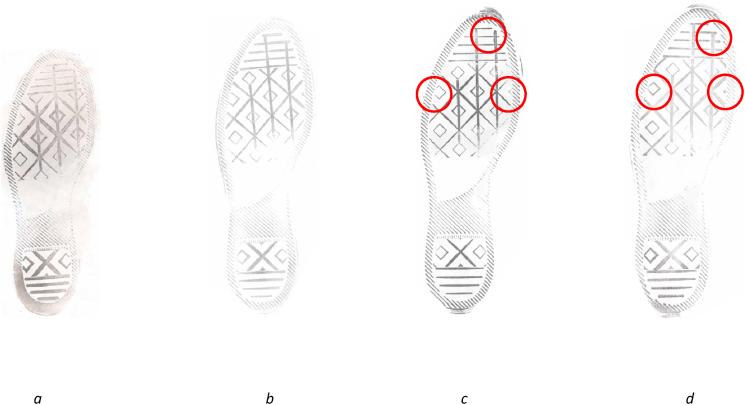
Converse All-Star (referenced as Converse 5 in the NFRC) shows configurational differences where the diamond lattice meets the edges and logo box. Fig. 2a shows a UK size 3, Fig. 2b a UK size 7 and Fig. 2c and d both show UK size 8s. The configuration on the UK size 7 (Fig. 2b) and one of the UK size 8s (Fig. 2c) are similar, whereas the configuration of the two size 8s (Fig. 2c and d) differs slightly as can be seen most clearly in the circled areas.

The *size* relates to the overall dimensions of the undersole of the shoe and is related to the manufacturer's shoe size [53], and *configuration* relates to the specific arrangement of the components of the pattern in relation to each other and to the edges of the undersole [43]. This can vary between or within sizes (as shown in Fig. 2).

As with pattern type, for the other class characteristic of size & configuration, the numerator can be assumed to be one. There may be some circumstances where the size & configuration may appear to differ between the mark and the footwear even if the footwear had made the mark, for example if there is movement in the mark or the mark is in a contaminant or on a surface which has led to expansion, contraction or distortion of the mark. However it is fair to assume that, for the most part, the probability that the shoe and mark would match is one (i.e. certain) if the mark had indeed been made by the shoe [50]; the method for updating the numerator and denominator if there are explicable differences is discussed further in section 5.4. Determining the denominator is less straightforward. The crime scene mark or test impression of the suspect's footwear can be compared to a sample of prints of the same pattern type from a reference collection [33] and an assessment made of whether a particular size or configuration of the pattern could be excluded as having made the mark. This can lead to a size range within which the shoe which made the mark could be expected to fall. Dependent on the quality and extent of the mark, this could include marks of only one particular size or even one particular mould or could extend to a wide range of sizes [44,55]. The reference collection or database can then be used to provide a relative frequency of this size or range of sizes within the considered population [50]. For example, if it is found that the shoe responsible for having made the mark could be expected to fall in the range of size 8-9, then one would determine the relative frequency of sizes 8, 8.5 and 9 within the wider population on the database [33].

### 2.4 Wear

**Fig. 3:**
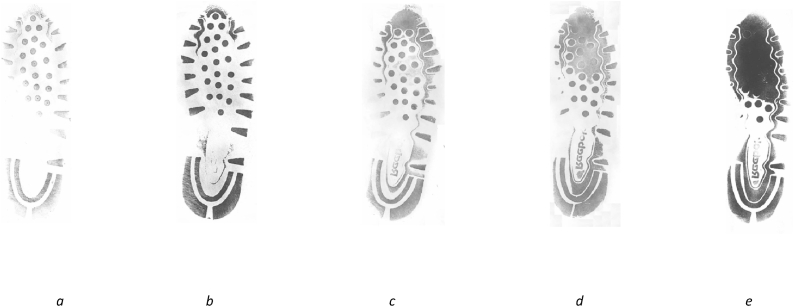
Reebok Classic (referenced as Reebok 3 in the NFRC) showing the progression of wear from a new shoe in Fig. 3a, with pips still visible on the studs and fine bars at the heel, progressing to a very heavily worn shoe in Fig. 3e, where many of the studs and components of the outer sole have worn away entirely. All shoes shown are a UK size 8, but are not the same shoe.

*Wear* relates to acquired changes to the undersole caused by friction with the ground when walking, which erodes or otherwise changes the formation of the pattern components [43,45,53]. The undersole of a heavily worn shoe can be significantly different from its initial state, to the point where it may not be recognisable as the same pattern type [43], as shown in Fig. 3.

For wear, the process for determining both the numerator and the denominator is more complicated, as one might not automatically assume that this property would match even if the mark had been made by the shoe. Differences in time and factors such as the quality of the mark, the background surface and movement in the mark, can mean that there are significant differences between the mark and the shoe, even if the mark were made by the shoe [56]. The evaluation of these characteristics is therefore more dependent on the judgement and experience of the practitioner [47].

A reference database may still be used to provide the practitioner with information about how the footwear type in question may change as the shoe is worn [50]. An assessment can be made by comparing the mark or test impression with a representative sample of prints of that pattern type from the reference collection to determine the proportion which display a similar degree of general wear to that observed in the crime scene mark [57]. There may also need to be a consideration of whether or not the footwear responsible for making the crime scene mark may have acquired more wear in the period since the mark was deposited [44,55,57].

### 2.5 Damage (also know as Randomly Acquired Characteristics or RACs)

**Fig. 4:**
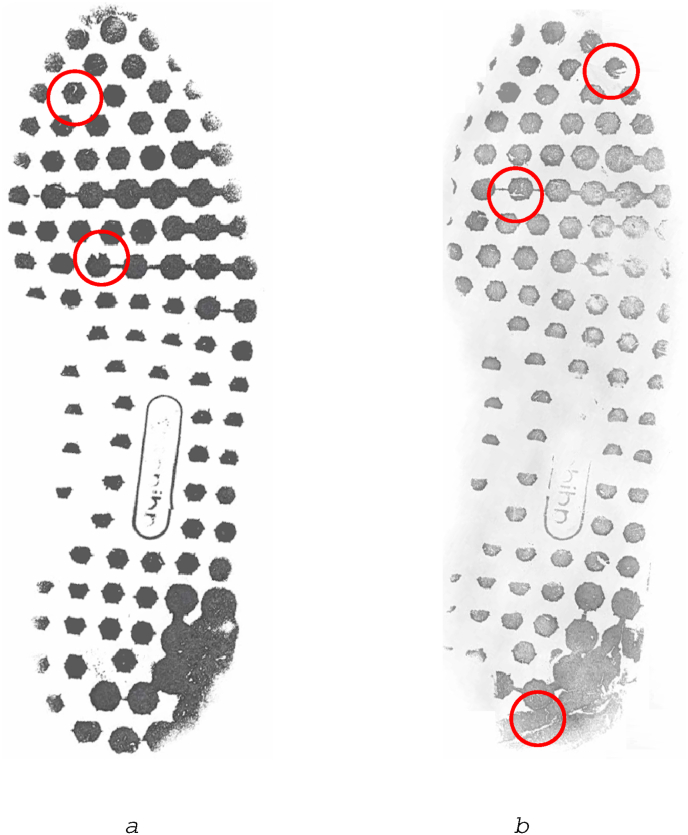
Adidas LA (referenced as Adidas 250 in the NFRC) showing damage features, circled. The features shown on Fig. 4a and in the ball of the foot area on Fig. 4b are scratches on blocks. The circled feature on the heel area of Fig. 4b shows damage associated with a heavily worn area of the shoe, known as wear-induced damage in the UK.

*Damage* refers to small or gross features on the undersole of the shoe, caused by tearing, scratching or other impairment to the sole surface [43,44], as shown in Fig. 4. In the US and elsewhere, this damage is often referred to as Randomly Acquired Characteristics (RACs) [58], but damage may have been caused deliberately by the owner of the shoe in order to attempt to change the appearance and inhibit comparison with crime scene marks; in the UK, therefore, this property is usually referred to as damage.

Assessing whether there are corresponding damage features and the significance of their presence or absence will be almost entirely based on the practitioner's expertise and experience [25,33]. Comparing the damage observed in a scene mark or on the sole of a shoe with examples of the same pattern from a database is likely to result in there being no or an extremely small number of examples with the same arrangement of features [59]. It is also difficult to establish from a test impression or image in a database whether a feature is due to damage or is an artefact of the way the test impression was made, a contaminant or a mould feature. A number of methods have been explored and suggested for the evaluation of corresponding damage features observed in a scene mark and considered footwear. Stone [60] suggested that a possible approach would be to divide the entire area of the shoe sole into a grid and assign a probability of a pinpoint feature occurring in each location, then multiply this probability for each feature observed. However, the probability becomes extremely small very quickly: Stone suggested that the sole would be divided into a grid of 16000 1 mm^2^ squares, which would result in only 2 features giving a probability of approximately 1 in 256 million. This approach is not generally adopted in the UK, due to this probability seeming too high, but also because it does not take into account the fact that the features may not be independent of each other, that some areas of a shoe sole are more likely to become damaged than others [61] or that the occurrence of damage may be dependent on the pattern type of the shoe [62,63], all of which would impact on the assessment of the significance and the validity of multiplying the probabilities.

The examiner will usually consider, based on their experience and judgement [47], how significant each observed corresponding feature is. When considering damage, an examiner is looking primarily at whether or not the features visible in the mark correspond with or differ from those observed on the suspect's footwear. Where there is correspondence, they will consider whether the shape is characteristic or not; for example a pinpoint feature is less significant than a feature with an unusual shape. Where there are differences or features are not observed in both the mark and the shoe, they will consider whether or not these differences can be explained by factors such as time delay, the quality of the mark or movement in the mark which may obscure detail. Where there are numerous features or one individual feature is distinctive in terms of its size, shape or orientation, the examiner may consider that the correspondence indicates that the mark could only have been made by the considered shoe and no other and will therefore give the opinion that there is a conclusive link between the mark and the shoe [56]. This “leap of faith” [64,65] is clearly at odds with the more structured approach used for evaluating other aspects of the comparison and leads to a two-tier system for evaluative opinion. There is a lack of empirical research to demonstrate fully the validity of the perception of uniqueness [28,66]; in one study, 39 pairs of shoes of the same make and model worn by the same person were compared [67]. Wilson concluded that it was possible to distinguish between the shoes based on random characteristics on the soles; whilst this may demonstrate that features are rarely repeated and therefore have some evidential significance, the study does not provide sufficient data to allow for the categorical identification of footwear impressions based on corresponding damage features. Nevertheless, this is still a conclusion which many forensic footwear examiners still report, both in the UK and internationally.

### 2.6 Overall LR

Once the LR for each characteristic has been established, they are multiplied together as shown in equation (1). An overall LR is then derived for the footwear comparison overall. This can then be commuted to a verbal expression for the strength of support for the hypothesis given by the evidence [33]. It should be noted, however that, as discussed in more detail in Section 4, multiplying the separate values for the LR for each characteristic together assumes independence between these properties which may not be the case.

## 3 Case study

**Fig. 5:**
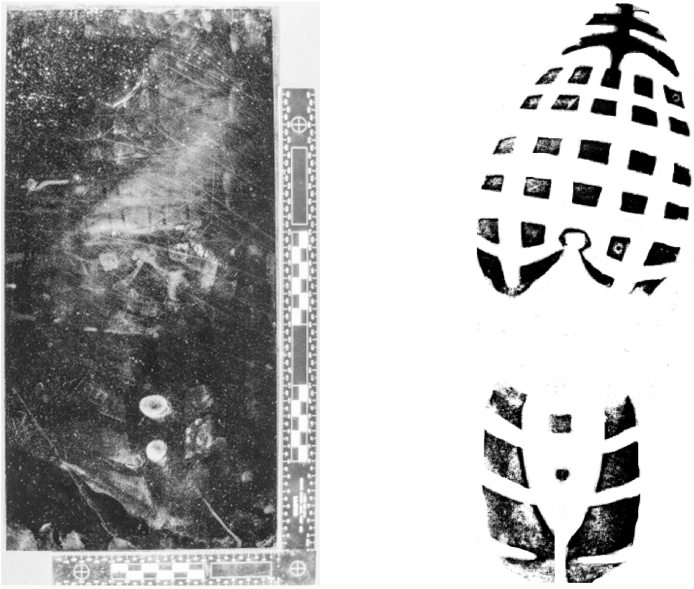
Fig. 5a: Black gel lift of a footwear mark, enhanced with aluminium fingerprint powder, recovered from the scene of a crime and photographed to scale; and Fig. 5b Reference example of pattern coded as Nike 189 in the National Footwear Reference Collection (NFRC).

This case study, based on a burglary that took place in the UK provides an example of how reference data can be used to interpret footwear evidence. A residential burglary occurred at an address in the north of England in 2013, during the course of which the offender stood on a kitchen work surface. CSI subsequently attended the crime scene and located a footwear mark, which they enhanced with aluminium fingerprint powder and lifted using a black gel lift. The black gel lift was submitted to the footwear unit where a photograph was taken with a scale (Fig. 5a). The pattern type of the mark was identified from the NFRC as Nike 189, which is one of the more frequently encountered undersole patterns in the UK (Fig. 5b).

Some time later, a suspect was identified and footwear recovered on arrest, which was submitted to the footwear unit for comparison to the crime scene mark. The suspect's footwear was found to correspond with the mark. To determine the significance of this “match” the practitioner considered the relevant frequency of the properties of the crime scene mark in the reference database.

The force in question maintains a footwear reference collection comprising prints of footwear encountered through casework or taken from detainees in custody. From data taken from this, the relative pattern frequency was estimated: in the 12 months prior to the offence being considered, the reference database recorded 11315 entries. Of these, 577 were of the Nike 189 pattern, giving a LR of 19.6 for pattern (LR = 11315/577).

Physical reference samples from the footwear reference collection were then considered. Examples of different sizes of the pattern type from the footwear reference collection were compared with the mark, and the mark was found to correspond with a size 11 shoe; all other sizes could be excluded. Of the 577 Nike 189 patterns seen in the previous 12 months, 30 of them were recorded as being size 11, giving a LR of 19.2 for size (LR = 577/30).

The 30 size 11 Nike 189 reference samples were retrieved from the footwear reference collection and compared with the crime scene mark, considering the degree and distribution of wear. Of the 30 examples of this pattern and size encountered in the previous 12 months, only 2 of them were found to correspond in terms of the degree and distribution of wear. All other examples were either too heavily worn, or not worn enough. This gives a LR for wear of 15 (LR = 30/2).

Overall therefore, the LR for the correspondence can be calculated as follows:

LR=P×S×W

LR=19.6×19.2×15

LR=5644.8

**Fig. 6:**
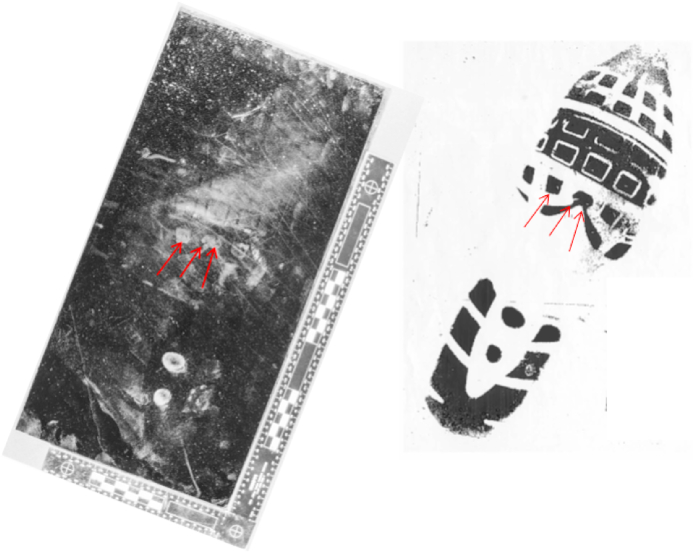
Crime scene mark and print taken from the suspect's shoe showing 3 corresponding features.

Damage has not been included in this calculation. In this example, the crime scene mark has three features visible, shown in Fig. 6 by the red arrows. These were also visible in the print taken from the suspect's shoe. It can be difficult to produce a LR value for damage. In this case, if the 577 examples of a Nike 189 were examined, it is unlikely that one would correspond with the mark in terms of damage if there were more than 2 or 3 features.

Instead, as described in section 2.5, the practitioner will make a subjective assessment of how likely they think it is that the corresponding features would be observed if the mark has been made by another shoe and the correspondence has occurred by chance, based on the number, position, size and shape of the damage features.

In summary, therefore, during a footwear mark comparison, each characteristic of the crime scene mark and suspect's footwear are independently evaluated, using reference data where available, to determine an indicative LR for each which are then multiplied to give an overall LR for the footwear mark match. Difficulties in determining a LR for damage leads to the use of a more qualitative and subjective assessment of the significance of correspondence for this property. The overall indicative LR is communicated as a verbal strength of support; where it is the opinion of the practitioner that the overall correspondence would only be observed if the mark was made by the suspect's footwear and no other, this may be reported as categorical evidence, rather than as a strength of support derived from the LR.

## 4 Weaknesses in this method

There are a number of weaknesses in the method described in section 2. Firstly, there may be issues with the figures used, which are not absolute [39]. For pattern, it has already been mentioned that relative frequency data could be obtained from either manufacturer or sales data, or a reference database held by a forensic unit, with the latter being the preference for footwear practitioners due to its relevance to criminal investigations [68,69]. However, there may be geographic differences which could mean that national frequency data does not reflect the local position [50,69]. Currently, not all police forces use the NFRC or populate its database with their observations; those forces which do, do not share a standard policy for which crime types to send CSI to or to recover crime scene footwear marks from, nor for which detainees should have their footwear sampled in custody [6]. Therefore, the data available is skewed to those police forces who do upload their information and is not balanced across those that do. Local data will reflect these inequalities, although it may better reflect the demographic and pattern frequencies in that force area [70].

The problem is exacerbated when a new or rare pattern is encountered [9,39]. A new pattern may be seen for the first time and considered to be of high significance as a result. However, a number of weeks later, this pattern may be one of the most frequently encountered. This issue can be mitigated to some extent by setting an upper limit for the LR for a new or rare pattern [50] but for those patterns which subsequently become popular, this may still lead to overestimates.

New and rare patterns will have little or no data associated with them, unless data is available from manufacturers [39]. In addition to the issue mentioned above, where this could potentially lead to pattern frequency assessments overestimating the significance of a pattern match, there is also less information available to evaluate how the pattern changes with size or wear. A dearth of reference examples will require the practitioner to use experience and professional judgment when considering how a pattern may vary [9]. However, lack of empirical evidence may lead to incorrect assumptions or ignorance of properties of a pattern such as specific characteristics of certain sizes or areas where a sole is more prone to wear. Reliance on professional judgment may also lead to differences of opinion and potential legal challenges [71,72].

Even where there is an abundance of data, demonstrating variation between sizes and moulds and indicating how a pattern changes with wear, the information is still incomplete and can provide an indicative figure only. Data is specific to the time and location [69], dependent on factors such as the population in the area where the reference samples were collected, the sampling policy of the organisation and even such ephemeral factors as seasons or fashion [70]. The popularity of a pattern can vary between countries, police force regions or even areas within a police force. Furthermore, when a practitioner accesses the reference data to inform the interpretation of the findings, this is done manually. The volume of data for a frequently encountered pattern makes it impractical, if not impossible, to assess every reference print, therefore a representative sample of reference prints is used to provide an indication of the frequency of occurrence of a characteristic of the pattern and therefore the significance of the correspondence between the crime scene mark and a suspect's footwear. Therefore, even where there is a wealth of data, an exact value for the LR is not produced, but an indicative figure only [50].

Finally, and possibly the most significant weakness of the method is the assumption of independence between the properties of the footwear.

In the formula

**Fig. 7:**
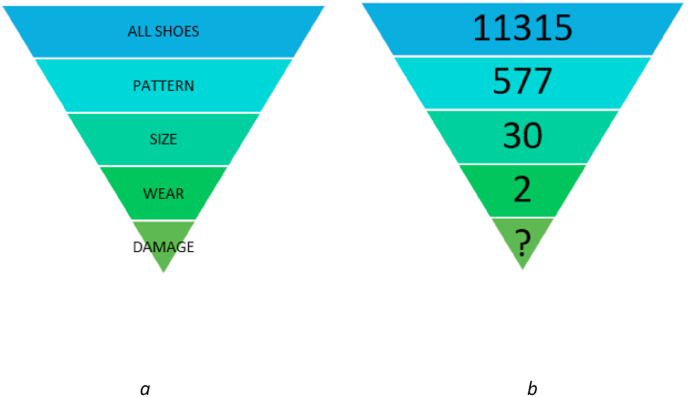
Fig. 7a: Using reference data, the number of shoes that can be included as a potential source of a crime scene footwear marks can be reduced from **All Shoes** to just those of the correct **Pattern**, to those of the correct **Pattern** and **Size**, then those of the correct **Pattern**, **Size** and **Wear** and, ultimately, only those shoes of the correct **Pattern**, **Size** and **Wear** which also display corresponding **Damage**. Fig. 7b: An example with figures using the approach shown in Fig. 7a. The database contains 11315 footwear records for the period in question. Of these, 577 are the correct **Pattern,** 30 of the 577 correspond in terms of **Size** and 2 of the 30 correspond in terms of the degree and distribution of **Wear**. Therefore, the population of shoes that can be considered as a potential source is reduced from 11315 to only 2.

LR=P×S×W×D

probability theory requires that the LR for each property is independent of the others in order to multiply them. However, this may not be the case: a pattern may only come in certain sizes, making size dependent on pattern. Furthermore, patterns may wear or become damaged in a particular manner due to the shape of the pattern components or material that the undersole is made from [25]. When there is a large collection of reference data for a pattern, this lack of independence can be mitigated by selecting a sample of reference prints and reducing the number for each property by excluding those which do not correspond with the footwear mark, as shown in Fig. 7.

## 5 Bayesian Networks

The aim of this paper is to determine whether the existing method of footwear analysis can be improved by developing a Bayesian Network tool that builds on existing data and knowledge to produce a more objective, consistent and transparent method for evaluating footwear evidence.

### 5.1 Method

To explore the appropriateness of a Bayesian Network for evaluating footwear evidence, a simple model was built using the 2010 Court of Appeal ruling R v T [7].^1^

A summary of the case details follows. On the 4th of July 2006, a male received a fatal gunshot to the thigh at an address in Manchester.^2^ Footwear marks were recovered from the crime scene and compared with shoes recovered from a suspect about 15 weeks after the offence [7]. The footwear corresponded in terms of the pattern, a Nike Air Max, which is frequently encountered in forensic examinations, and in terms of the size and configuration, which was UK size 11. The footwear was more worn than was observed in the mark and there were apparent damage features observed in the crime scene mark which were not present on the soles of the footwear and, conversely, damage on the soles of the footwear which was not observed in the mark. However, this was explained away by the scientist as being expected given the significant time delay.

At the initial trial, the scientist gave evidence that in his opinion there was “moderate scientific evidence” to support the view that the mark from the crime scene had been made by the suspect's footwear. The appeal court [7] reported that the scientist reached this opinion using figures from the database at the Forensic Science Service, which showed that 20% of the shoes encountered in casework were of the same pattern. National sales figures produced by the footwear industry indicated that 3% of shoes sold in the UK were size 11. However, the scientist testified that he modified this to allow for a number of factors including limitations of the crime scene mark, the existence of counterfeit shoes and the vagaries of the manufacturing process whereby the same size sole can be used for shoes of different sizes. The scientist therefore used a LR of 5 for the pattern and 10 for the size.

The scientist then explained that, although the degree and distribution of wear on the footwear differed from that observed in the crime scene marks, he would still expect to be able to exclude half of the shoes of this pattern and size based on wear, giving a LR of 2 for wear. As there were differences in terms of damage and due to the time delay, the scientist testified that he would be able to exclude very few additional shoes that had not already been excluded on the basis of pattern, size and wear, therefore he assigned a value of 1 for the LR for damage. The scientist then went on to explain that, as there were differences, this finding was more likely if the shoes had not made the marks, therefore he moderated the LR for this aspect of the evidence to be somewhat less than one.

As explained in Section 2, he then multiplied the LR for the characteristics together as follows^3^:

LR=P×S×W×D

LR=5×10×2×<1

LR=∼100

On cross-examination, questions about sales figures in the UK were asked, with the scientist giving an estimated figure of around 42 million pairs of shoes sold each year, and an estimate of around 300 million pairs of sports shoes over the previous 7-8 years. The scientist said that the distribution figure for this particular Nike pattern was approximately 786,000.

### 5.2 Building the Bayesian Network

**Fig. 8:**
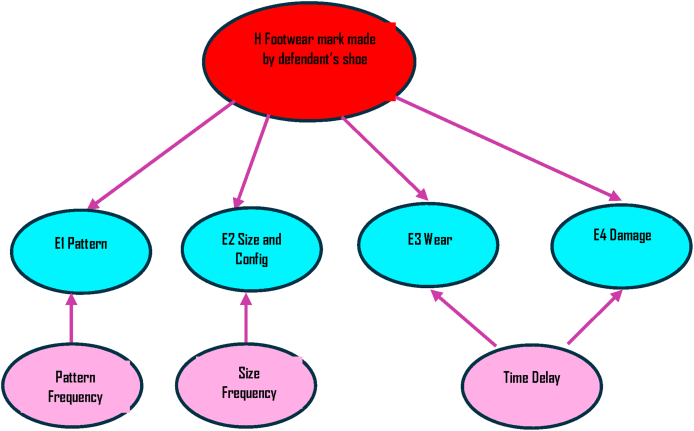
A simple Bayesian Network constructed based on the footwear evidence discussed in the R v T appeal court judgment.

To illustrate how a Bayesian Network model could address the problems associated with forensic footwear evaluation and provide a method that can be usefully deployed in operational forensic science we constructed a simple Bayesian Network (Fig. 8) with nodes for each of the properties of the footwear – *Pattern*, *Size & Configuration*, *Wear* and *Damage*. We included parent nodes for *Pattern* and *Size & Configuration,* each linked to the relevant frequency data for these variables, using a number of different databases. We also included a separate node to represent the time delay between the crime and the recovery of the suspect's footwear, which will have an impact on both *Wear* and *Damage* and may provide an explanation for differences. The target hypothesis in the Bayesian Network was that the footwear was made by the defendant's shoes and the alternative was that it was made by some other shoes. For the purpose of illustration in this model, the prior odds were set as 50/50 for these hypotheses.

The figures quoted in the appeal court judgment [7], both from the Forensic Science Service database and the sales figures, have been used to populate the CPT. For comparison, figures from a UK police force footwear database of prints taken in custody for the period 1999-2006 and figures from the National Footwear Reference Collection (NFRC) database of prisoner prints for the period 2010-2022^4^ were also considered. The data used is shown in Table 1.

**Table 1:** Data from four different sources used to test a Bayesian Network model based on the R v T case. Without access to the crime scene mark from the case, it is not possible to determine the proportion of footwear examples that would be considered to correspond in terms of wear therefore no value can be quoted for the UK Police Force or NFRC National Custody data; wear data would not be available from manufacturers as it relates to usage of the footwear rather than production. For all models the Forensic Science Service scientist's estimate of 50% is therefore used.

Database	Pattern frequency	Size frequency	Wear
Forensic Science Service data used by scientist	20%	10%	50%
Manufacturer details as quoted in the appeal hearing	786,000 out of 300 million = 0.26%	3%	N/A
UK Police Force custody data 1999-2006	1800 out of 67,215 = 3%	26 out of 525 = 5%	N/A
NFRC National Custody data 2010-2022	25,825 out of 707,337 = 4%		N/A

### 5.3 Sensitivity analysis

**Fig. 9:**
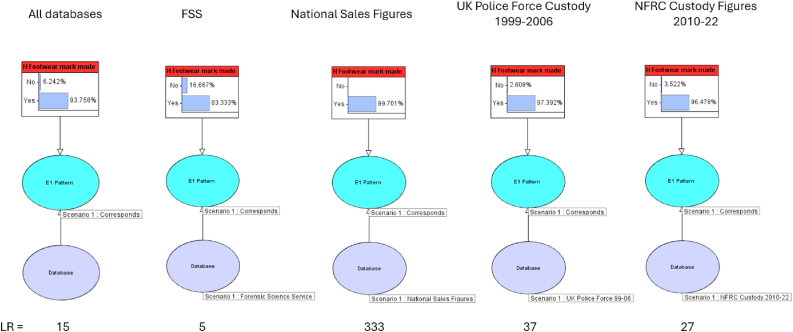
Sensitivity Analysis of the effect of different pattern frequency probabilities from different databases: a) All databases; b) Forensic Science Service (FSS) data quoted in the R v T judgment; c) national sales figures quoted in the R v T judgment; d) UK police force custody data; e) National Footwear Reference Collection (NFRC) custody data.

The Bayesian Network and CPT values were first used to undertake a sensitivity analysis to understand the effect of the figures from different databases for pattern frequency on the posterior probability and overall LR for the hypotheses (Fig. 9). This was done by selecting the data from each of the four different datasets in the Bayesian network and recording the posterior probability. For the purposes of this model, the prior odds were set as 50/50, but the standard practice is to quote the LR, allowing the fact-finder to set and update their own prior and calculate the posterior odds. Because the odds form of Bayes’ rule is:

PosteriorOdds=LR×PriorOdds

then the LR can also be calculated by taking the ratio of the posterior and prior odds.

Initially, the model was used with data from all four sources considered, using equal prior probabilities and not selecting for a particular database, therefore averaging across all databases. The posterior probability in this case was 93.8%, equating to a LR of 15, supporting the hypothesis that the footwear mark was made by the defendant's shoe. Whilst not undertaken for this paper, it would be possible to apply differential weighting to different databases to reflect their relevance to the circumstances.

Each database was then used separately and a sensitivity analysis undertaken, which shows that the UK police force and NFRC Custody figures are similar, providing posterior probabilities of 97.4% and 96.5%, equating to LR of 37 and 27 respectively, to support the hypothesis. The figures based on the FSS data are slightly lower, with posterior probabilities of 83.3% produced, equating to a LR of 5. However, a much smaller database was apparently used, with the R v T judgment [7] stating that the FSS database represented “approximately 0.00006% of all shoes sold in a year”; based on the figure of 42 million pairs of shoes sold every year quoted in the judgment, this would equate to approximately 25. This figure seems unlikely and it is probable that it is more likely to be around 2,500, which would equate to 0.006% of all shoes sold in a year or 0.00006 times the number of shoes sold.^5^ By comparison, as shown in Table 1, the UK police force custody database comprised approximately 67,000 records for the time period considered and the NFRC Custody figures comprised around 707,000 records. Therefore, it can be seen that the FSS figure may be less robust due to its being based on a significantly smaller database. Nevertheless, it is still in a similar range to the LR value from the UK police force and NFRC Custody data.

Conversely, the figure generated using the national sales figures produced a significantly higher value, giving a posterior probability of 99.7%, equating to a LR of 333. These are the figures which the defence suggested would be more accurate than the FSS database figures, but ironically are notably less favourable to the defendant. As explained in Section 2.1, the figure is higher due to the total sales figures for footwear in the UK including all footwear, including high heels, children's shoes, sandals and slippers, with a particular model of footwear being a smaller proportion of the overall population and therefore a chance match appearing less likely. The crime-related data from the FSS, UK police force and the NFRC however will predominantly comprise footwear worn by people suspected of committing crimes, with a preponderance of training shoes [52]. Within such databases, a particular pattern of training shoe will, relatively speaking, comprise a larger proportion of the population and therefore the significance attributed will be lower.

The similarity in the figures for all three crime-related databases, in particular the larger UK police force and NFRC ones, suggests that the database used does not have a significant impact on the LR or posterior probability, as long as the database used is relevant to the population being considered, in this case those suspected of committing a crime. The range of values could also be quoted, including minimum and maximum values for the LR [70]. However, it would provide a more coherent value if figures from sales databases, which are skewed due to the inclusion of data relating to footwear which is not relevant to the investigation of most crimes, were not used as part of this exercise. In this example, excluding the sales figure value, the maximum value for the LR for pattern alone would be 37 and the minimum value 5.

### 5.4 Accounting for differences

Another difficulty encountered by footwear examiners and contributing to the misunderstanding in the R v T case is how to account for differences between the crime scene mark and the compared footwear where such differences do not necessarily mean that the footwear did not make the mark and can be excluded. Manufactured characteristics such as pattern must correspond if the mark has been made by the footwear: if the pattern differs then the footwear cannot have made the mark and must be excluded. This applies to a certain degree for size & configuration, although there may be some conditions where the size or configuration may appear different, for example if there is movement in the crime scene mark [73] or if an impression is made in a substrate such as mud which may contract or expand over time [44].

More frequently observed are differences in the degree and distribution of wear and acquired features such as damage. As with size & configuration, some conditions may lead to apparent differences between the acquired features observed in the crime scene mark and those present on the sole of the compared shoe: movement can smear blocks or obscure fine features. In addition, if a period of time has elapsed since the crime was committed, then the shoes may have acquired more wear in the intervening period [44]. In such cases these differences can be “explained away” [16,74]. The explanation that the shoes have become more worn since the crime accounts for the observed difference between the shoes and the mark, and thus makes it less probable that another shoe made the mark instead [16].

In some cases, the differences between shoes and mark are more pronounced than expected in the period of time. Nevertheless, uncertainty about the use of the footwear in the intervening period and a lack of research into how different footwear types vary with wear may preclude a conclusive exclusion of the footwear [44].

As a means to calculate the LR, the probability of observing corresponding features if the mark was made by the shoe approaches 1. In a case where the wear differences can be explained and might even be expected, then the probability would still be close to 1. However, if there are unexpected differences or differences that are more pronounced than might be expected, then the probability of these observations, and hence the numerator in the LR calculation, would be less than 1. However, it is not clear how to calculate a reasonable figure for this, and how much less than 1 the probability would be.

In the R v T hearing, the scientist reported that he had used a figure of LR = 2 for wear, because the differences would be expected due to the time delay of 15 weeks and he would still expect to exclude half the training shoes of this pattern type on the basis of wear [7]. However, there were also features visible in the marks from the crime scene which suggested that the responsible shoes bore damage to the undersoles. This damage was not present on the footwear [7]. Again, the scientist considered that there may be a number of explanations for these differences and that the figure would still be close to 1. However, to account for the fact that the absence of the damage made it somewhat less likely that they had made the marks, he used a value of <1. Using a Bayesian Network would allow a practitioner to introduce estimates of the probability that the damage would differ into the CPT, leading to a more robust indication of the impact of the differences on the evidential strength and overall odds.

### 5.5 Simple Bayesian Network using quantitative and qualitative data

The CPT for the simple Bayesian Network shown in Fig. 8 was populated using quantitative data from a number of databases for *pattern* and *size & configuration*, as shown in Table 2, Table 3 and qualitative data for *wear* and *damage*, as shown in Table 4, Table 5, to model a number of different scenarios. The probability data for *wear* and *damage* was based on qualitative judgments of a forensic footwear expert based in a UK police force with over 25 years’ experience, using their professional judgment, knowledge and casework experience.

**Table 2:** Conditional Probability Table (CPT) for pattern frequency for Nike Air Max (node E1 Pattern) used for the Bayesian Networks in Fig. 10, Fig. 11, Fig. 12, with the prosecution hypothesis, H, being “Footwear mark made by defendant's shoe” and alternative hypothesis, ∼H, being “Footwear mark made by some other shoe”. The data for the Forensic Science Service and National Sales Figures are based on the figures quoted in the R v T judgment.

Footwear mark made by suspect's shoe	No				Yes			
Database	Forensic Science Service	National Sales Figures	UK Police Force 99-06	NFRC Custody 2010-22	Forensic Science Service	National Sales Figures	UK Police Force 99-06	NFRC Custody 2010-22
Differs	0.8	0.997	0.9732	0.9635	0.0	0.0	0.0	0.0
Corresponds	0.2	0.003	0.0268	0.0365	1.0	1.0	1.0	1.0

**Table 3:** Conditional Probability Table (CPT) for size 11 frequency (node E2 Size and Config) used for the Bayesian Networks in Fig. 10, Fig. 11, Fig. 12. The data for the Forensic Science Service and SATRA data are based on the figures quoted in the R v T judgment.

Footwear mark made by suspect's shoe	No			Yes		
Database	Forensic Science Service	SATRA	UK Police Force 99-06	Forensic Science Service	SATRA	UK Police Force 99-06
Differs	0.9	0.97	0.9505	0.0	0.0	0.0
Corresponds	0.1	0.03	0.0495	1.0	1.0	1.0

**Table 4:** Conditional Probability Table (CPT) for wear (node E3 Wear) used for the Bayesian Networks in Fig. 11, Fig. 12. The probabilities for correspondence or difference given different time delay periods have been assigned using a qualitative analysis rather than based on quantitative data.

Footwear mark made by suspect's shoe	No					Yes				
Time Delay	None	Up to 1 month	1 to 3 months	3 to 6 months	Over 6 months	None	Up to 1 month	1 to 3 months	3 to 6 months	Over 6 months
Differs	0.9	0.9	0.9	0.9	0.9	0.0	0.1	0.3	0.5	0.7
Corresponds	0.1	0.1	0.1	0.1	0.1	1.0	0.9	0.7	0.5	0.3

**Table 5:** Conditional Probability Table (CPT) for damage (node E4 Damage) used for the Bayesian Networks in Fig. 11, Fig. 12. The probabilities for correspondence or difference given different time delay periods have been assigned using a qualitative analysis rather than based on quantitative data.

Footwear mark made by suspect's shoe	No					Yes				
Time Delay	None	Up to 1 month	1 to 3 months	3 to 6 months	Over 6 months	None	Up to 1 month	1 to 3 months	3 to 6 months	Over 6 months
Differs	0.7	0.7	0.7	0.7	0.7	0.0	0.1	0.2	0.4	0.6
Corresponds	0.05	0.05	0.05	0.05	0.05	0.7	0.5	0.3	0.2	0.1
No damage visible	0.15	0.15	0.15	0.15	0.15	0.15	0.15	0.15	0.15	0.15
Mixed correspondence	0.1	0.1	0.1	0.1	0.1	0.15	0.25	0.35	0.25	0.15

For the manufactured characteristics, the figures shown in Table 2, Table 3 from the Forensic Science Service, manufacturer and SATRA data are those quoted in the R v T judgment [7], with UK police force data taken from the period 1999-2006; and the NFRC Custody data taken from the period 01/01/2010-04/02/2022.

We assigned probabilities to the CPT for wear and damage based on qualitative judgment, based on the knowledge and experience of a forensic footwear expert based in a UK police force, rather than quantitative data, as shown in Table 4, Table 5. However, further work could include using the Bayesian Network to incorporate different values or views of other forensic footwear experts to show how sensitive our results are to changes in the values in the CPTs; this again highlights the power and transparency of this method. For wear and damage, the effect of time delay on these properties was also incorporated, using time delay periods broken into five categories: *none*, *up to* *1 month*, *1 to* *3 months*, *3 to* *6 months* and *over* *6 months*. When determining the values to be used in the CPTs for wear and damage, the forensic footwear expert took other factors into account, as well as the time delay, including the impact of the quality of the crime scene mark, movement in the mark, the circumstances under which the mark was made and factors such as the substrate or contaminant, all of which could produce effects in the crime scene mark which would make the wear appear different from the responsible shoe.

If the footwear mark had not been made by the defendant's footwear, we judged that it was more likely that the wear would differ; the probability that the wear would differ in these circumstances would be independent of time delay and would therefore be the same across each period. For these circumstances, therefore, probabilities were assigned as 0.9 that the wear would differ and 0.1 that it would correspond, as shown in Table 4.

When considering the probabilities to be assigned if the footwear had been made by the defendant's footwear, we judged that, if there was no time delay and the wear differed, this would mean that the shoe could not have made the mark. However, as the time delay increased, the probability of the wear differing would increase, although, even with a long time delay, such as *over* *6 months*, there would still be a likelihood of the wear corresponding, for example if the footwear had not been worn in the intervening period. Therefore, a probability of 0.0 was assigned to the CPT for the wear differing with *no time delay*, increasing to 0.7 with a time delay of *over* *6 months*.

For damage, four possible observations were included in the CPT, as shown in Table 5: *differs*, *corresponds*, *no damage visible* and *mixed correspondence* (i.e. there is damage which corresponds and damage which differs). As for wear, if we consider the probability of the observed damage correspondence if the footwear mark had not been made by the defendant's footwear, it is more likely that the damage would differ than correspond and this would be independent of the time delay. In this scenario, probabilities were assigned as 0.7 that the damage would *differ*, 0.05 that it would *correspond*, 0.15 that there would be *no damage visible* and 0.1 that there would be *mixed correspondence*; these probabilities were assigned for all time delay periods.

When considering the probabilities to be assigned if the footwear had been made by the defendant's footwear, we judged that, if there was no time delay and the damage differed, this would mean that the shoe could not have made the mark.^6^ As the time delay increases, however, the probability of the damage differing would increase, although, as for wear, even with a long time delay, such as *over* *6 months*, there would still be a small chance of the damage corresponding. The probability for damage differing was therefore set to increase with increasing time delay from 0.0 for *no time delay* up to a value of 0.6 for a time delay of *over* *6 months*.

For all of the time delay categories, including *no time delay*, the same probability, 0.15, was assigned for the observation *no damage visible*, as we judged that this is dependent not on the time delay, but on factors such as the quality and extent of the footwear mark.

Demonstrating the opposite effect to the chance of the damage differing, probabilities for *corresponds* were set to decrease with increasing time delay; probabilities of 0.7 for *no time delay*, decreasing to 0.1 with a time delay of *over* *6 months* were therefore assigned to the CPT for the damage corresponding.

Finally, we considered that it was most likely that there would be good correspondence with *no time delay* and no correspondence with *over* *6 months* time delay. We judged that, dependent on the quality of the mark and the activity that had taken place involving the footwear, there was a chance that there could be some differences in terms of damage for both of these time delay periods, therefore, *mixed correspondence* was given a probability of 0.15 in both these scenarios. However, we judged that with a significant but shorter time delay, we would expect that there would be some damage which continued to correspond, but some that would have been lost or gained in the intervening period, leading to some differences. Therefore, probabilities of 0.25 were given for time delays *up to* *1 month* and *between 3-6 months* and of 0.35 for a time delay of *1-3 months* for *mixed correspondence*.

#### 5.5.1 Scenario 1: corresponding manufactured characteristics

**Fig. 10:**
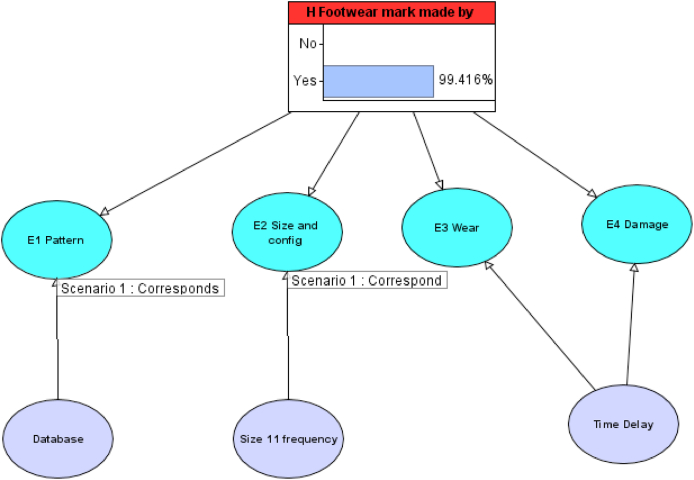
Simple Bayesian Network as shown in Fig. 8, showing the observation of corresponding manufactured characteristics as outcomes.

Firstly, only corresponding manufactured characteristics were shown as outcomes, using the CPT values shown in Table 2, Table 3. In this scenario, no specific database for either pattern frequency or frequency of size 11 was selected, therefore the result is based on the combination of all databases used. Selecting the outcome for both pattern and size as corresponding, this resulted in a posterior probability of 99.4% or a LR of 171,^7^ as shown in Fig. 10, which would equate to moderately strong support on the verbal evidential scale.

#### 5.5.2 Scenario 2: differing acquired characteristics with no time delay

**Fig. 11:**
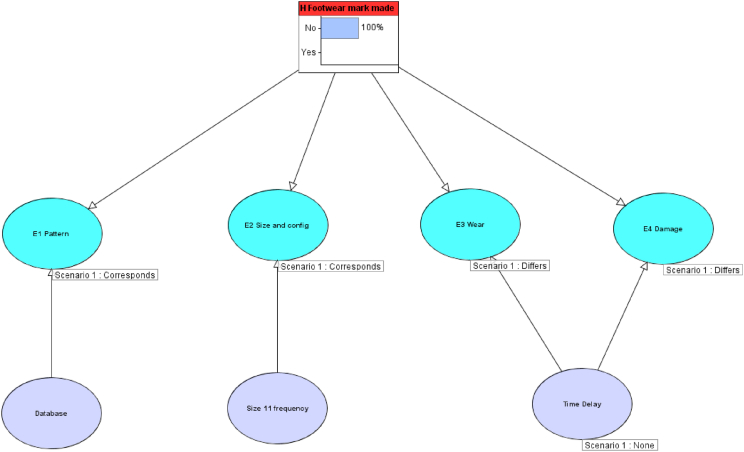
Simple Bayesian Network as shown in Fig. 8, Fig. 10, updated to include the observations that the wear and damage differ but there is no time delay as described in scenario 2.

The Bayesian Network was updated to include the observations that the wear and damage differ but there is no time delay, as shown in Fig. 11. As detailed above, it would be expected that, in this scenario, this would mean that the shoe could not have made the mark and probabilities of 0 were assigned both for the wear and the damage differing (Table 4, Table 5). Therefore, the posterior probability is 100% in favour of the defence proposition, that the footwear mark was made by footwear other than the defendant's; this would be reported as an exclusion of the considered footwear.

#### 5.5.3 Scenario 3: wear and damage differ with time delay

**Fig. 12:**
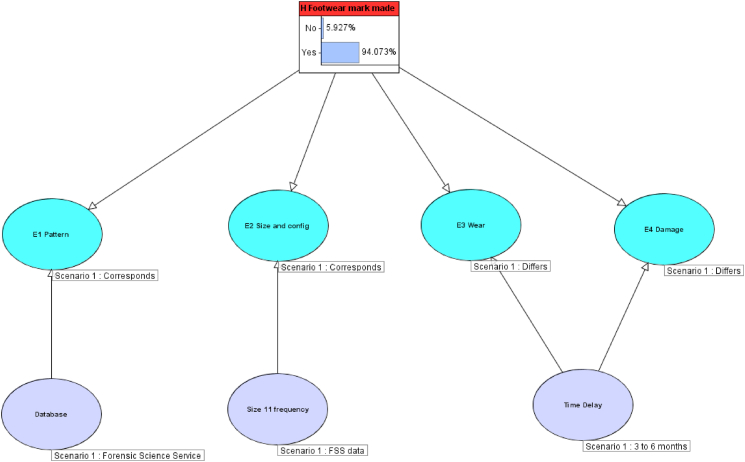
Simple Bayesian Network as shown in Fig. 8, Fig. 10, Fig. 11 showing pattern and size correspondence, with wear and damage differing, but a 15 week time delay noted.

Using the data in the CPT as shown in Table 4, Table 5, the observations were again updated, as shown in Fig. 12, to represent the findings reported in the R v T appeal court judgment: pattern and size correspond, wear and damage differ but there is a 15 week time delay. The differences in wear and damage are what might be expected given the time delay and can therefore be explained. This results in a posterior probability of 94% or a LR of 16 in favour of the prosecution hypothesis. This equates to moderate support on the verbal evidential scale, which is the same category as that reported in the R v T case. However, the use of the Bayesian Network gives a more transparent and robust LR, with a logical basis for reducing the LR to include the differences.

## 6 Discussion

The Bayesian Network presented here (Fig. 8, Fig. 10, Fig. 11, Fig. 12) provides a simple model for evaluating a straightforward footwear case using both quantitative data from a database and qualitative data based on the practitioner's experience and expectations. We propose that a Bayesian Network model could lead to a more robust and representative evaluation of the forensic findings. Using such a tool could provide a transparent and repeatable method which can help to communicate to other forensic scientists and relevant legal professionals how a conclusion was derived. The ability to evaluate both qualitative and quantitative probabilities and use the Bayesian Network as a graphical presentation in conjunction with numerical conclusions, verbal conclusions or both will also demonstrate that forensic findings are based on a logical and balanced assessment of the findings in the context of the prosecution and defence hypotheses rather than a definitive and empirical result or score [75].

Although the use of a LR to evaluate forensic findings in forensic footwear cases is a well-adopted method used in most of the UK and Europe, issues at court show that this method is not well understood by those outside the forensic community.

Differences between databases may lead to inconsistencies between practitioners and hence challenges from the defence and confusion at court when differing LRs are presented. As shown in section 5.3 of this paper, sensitivity analysis of different databases can demonstrate the range of possible values for the LR enabling the reporting of maximum and minimum values for the LR [70]. The most relevant database could be used to inform the expert's opinion on the strength of the evidence, but the range of values could also be quoted if other databases are available. Alternatively, data from different databases could be combined to provide a LR based on all available databases; this option might be particularly appropriate if there is limited information about the population that the true offender was a member of [76]. A conservative approach would be to use different databases to calculate the LR and then use the lowest LR, providing the strength of evidence that is the lowest. Sensitivity analysis and modelling of different outcomes based on different or combined databases can all be undertaken more easily with the use of a graphical modelling tool. Further work could also be undertaken to develop sensitivity analyses where differential weighting is applied to databases based on their relevance to the case circumstances or to consider data for other properties such as size, potentially weighting this to consider factors such as the gender of the wearer or offender.

If an operational tool were to be developed, this could include a link to databases, such as the NFD or police force databases to populate CPTs directly, with the practitioner able to suggest variables such as the pattern type and date range. The simple model proposed here could also be extended to incorporate other information or evidence, and could support a pre-assessment of the submission to decide whether or not to proceed with a full examination.

A BN model could also allow for the use and combination of both database information and expert judgement, with findings presented in context, both with relevant case information and with other evidence in the case, to support fact finders' evaluation within the wider context of the case as a whole [77]. The simple Bayesian Network shown in Fig. 8 could be developed further to incorporate other forensic and non-forensic science evidence and information, to account for the practitioner's confidence in the observations and even by the court to combine the forensic findings with other evidence presented at court. Bayesian Networks are not routinely used in forensic science in the UK, possibly due to the difficulty in constructing the initial model [19]; a tool for practitioners to use, including templates which can be used to model different scenarios would support their increased use. Expansion of the simple model in this paper will hopefully encourage more use and support improved reporting of forensic evidence.

## 7 Conclusion

Given the developments of technology that are creating more opportunities to interrogate datasets and develop robust methods for harnessing the insight contained within such datasets there is an opportunity to consider the potential for developing a tool for practitioners to help with inference, strengthen evaluative opinion and provide a robust, transparent and understandable method to communicate the significance of forensic findings.

This paper presented an overview of current approaches to footwear analysis and interpretation in England and Wales and identified issues that can lead to challenges in forensic evaluation. A Bayesian Network model was developed and presented here that could address these problems, both in forensic footwear examination and also in the broader forensic science practice.

## CRediT authorship contribution statement

**Danyela Kellett:** Writing – original draft, Validation, Project administration, Methodology, Investigation, Formal analysis, Conceptualization. **David Lagnado:** Writing – review & editing, Validation, Supervision, Methodology, Conceptualization. **Ruth Morgan:** Writing – review & editing, Supervision, Conceptualization. **Sherry Nakhaeizadeh:** Writing – review & editing, Supervision, Conceptualization.

## Declaration of competing interest

A portion of this research is part of a PhD research project but none of the work submitted in this manuscript has been previously published.

The authors declare that they have no known competing financial interests or personal relationships that could have appeared to influence the work reported in this paper.

## Footnotes

1 AgenaRisk software [78] was used to produce the models.

2 Drug dealer ‘executed as children hid in fear’ in Moss Side house, court told [79].

3 Despite using a figure of “<1” for damage, the overall LR is quoted as “∼100” which suggests that the LR has been rounded up to 100 and therefore the evidence that undermines the prosecution has been discounted. However, the evidential strength was reported as “moderate support” which equates to a LR of 10-100, therefore this accommodates the fact that the LR would be less than 100, but this is still potentially misleading to anyone scrutinizing the calculations in the appeal.

4 The NFRC did not launch until 2010, so data is not available for the period used in the R v T judgment.

5 The size of the database used is quoted in Hancock et al. (2012) as 8,122, but it is not clear where this figure was obtained from.

6 Assuming that the damage features could be confirmed.

7 This is just for the footwear evidence in this case and uses prior odds of 50/50.

## References

[1] Wen Z., Curran J.M., Wevers G. (2023). Shoeprint image retrieval and crime scene shoeprint image linking by using convolutional neural network and normalized cross correlation. Sci. Justice.

[2] Park S., Carriquiry A. (2021). Quantifying the similarity of 2D images using edge pixels: an application to the forensic comparison of footwear impressions. J. Appl. Stat..

[3] Milne R. (2012).

[4] Alizadeh S., Jond H.B., Nabiyev V.V., Kose C. (2021). Automatic retrieval of shoeprints using modified multi-block local binary pattern. Symmetry (Basel).

[5] Lin E.-T., Speir J.A. (2024). Predicting image quality of forensic footwear impressions. Sci. Justice.

[6] FCN (2021).

[7] (2010). R V T [2010] EWCA Crim 2439.

[8] Bali A.S., Edmond G., Ballantyne K.N., Kemp R.I., Martire K.A. (2020). Communicating forensic science opinion: an examination of expert reporting practices. Sci. Justice.

[9] Georgiou N., Morgan R.M., French J.C. (2023). The shifting narrative of uncertainty: a case for the coherent and consistent consideration of uncertainty in forensic science. Aust. J. Forensic Sci..

[10] Fenton N., Neil M., Lagnado D.A. (2013). A general structure for legal arguments about evidence using Bayesian networks. Cogn. Sci..

[11] Taroni F., Aitken C.G.G., Garbolino P., Biedermann A. (2006).

[12] Garbolino P., Taroni F. (2002). Evaluation of scientific evidence using Bayesian networks. Forensic Sci. Int..

[13] Cruz N., Desai S., Dewitt S., Hahn U., Lagnado D., Liefgreen A., Phillips K., Pilditch T., Tešić M. (2020).

[14] Pearl J. (1988).

[15] Lagnado D.A., Fenton N., Neil M. (2013). Legal idioms: a framework for evidential reasoning. Argument Comput..

[16] Lagnado D.A. (2021).

[17] Samie L., Champod C., Hicks T., Delemont S., Castella V. (2025). Driver or passenger? Use of a Bayesian network for the evaluation of DNA results in a fatal car accident. Forensic Sci. Int. Genet..

[18] Taylor D., Biedermann A., Hicks T., Champod C. (2018). A template for constructing Bayesian networks in forensic biology cases when considering activity level propositions. Forensic Sci. Int. Genet..

[19] Uitdehaag S.C.A., Donders T.H., Kuiper I., Wagner-Cremer F., Sjerps M.J. (2022). Use of Bayesian networks in forensic soil casework. Sci. Justice.

[20] Juchli P., Biedermann A., Taroni F. (2012). Graphical probabilistic analysis of the combination of items of evidence. Law Probab. Risk.

[21] Vink M., Sjerps M.J. (2023). A collection of idioms for modeling activity level evaluations in forensic science. Forensic Sci. Int..

[22] Smit N., Lagnado D., Morgan R., Fenton N. (2016).

[23] de Koeijer J.A., Sjerps M.J., Vergeer P., Berger C.E.H. (2020). Combining evidence in complex cases - a practical approach to interdisciplinary casework. Sci. Justice.

[24] Spencer N.A., Murray J.S. (2020). A bayesian hierarchical model for evaluating forensic footwear evidence. Ann. Appl. Stat..

[25] Evett I.W., Lambert J.A., Buckleton J.S. (1998). A Bayesian approach to interpreting footwear marks in forensic casework. Sci. Justice.

[26] Keereweer I., Van Beest M., Van de Velde J.M. (2005).

[27] Majamaa H., Ytti A. (1996). Survey of the conclusions drawn of similar footwear cases in various crime laboratories. Forensic Sci. Int..

[28] Kellett D., Zolghadriha S., Morgan R., Lagnado D., Nakhaeizadeh S. (2024). Forensic footwear examination: a systematic review of the existing literature. Forensic Sci. Int..

[29] Banks D.L., Kafadar K., Kaye D.H., Tackett M. (2021).

[30] Fenton N., Neil M. (2012).

[31] Evett I., Cook R., Jackson G., Jones P., Lambert J. (1998). A model for case assessment and interpretation. Sci. Justice.

[32] Jackson G., Aitken C., Roberts P. (2015).

[33] ENFSI (2015).

[34] AFSP (2008).

[35] SWGTREAD (2006).

[36] SWGTREAD (2013).

[37] Forensic Science Regulator (2021).

[38] President’s Council of Advisors on Science and Technology (2016). *Forensic Science in Criminal Courts: Ensuring Scientific Validity of Feature-Comparison Methods*.</div>.

[39] Kruse C. (2013). The Bayesian approach to forensic evidence: evaluating, communicating, and distributing responsibility. Soc. Stud. Sci..

[40] Cook R., Evett I.W., Jackson G., Jones P.J., Lambert J.A. (1998). A hierarchy of propositions: deciding which level to address in casework. Sci. Justice.

[41] Thompson W.C., Thompson William C. (2018). Seton Hall Law Review 773 MLA 9th ed..

[42] SWGTREAD (2006).

[43] College of Policing (2021).

[44] Bodziak W.J. (2000).

[45] Hicklin R.A., McVicker Brian C., Parks C., LeMay J., Richetelli N., Smith M., Buscaglia J., Perlman Rebecca S., Peters Eugene M., Eckenrode Brian A. (2022). Accuracy, reproducibility, and repeatability of forensic footwear examiner decisions. Forensic Sci. Int..

[46] Venkatasubramanian G., Hegde V., Lund S.P., Iyer H., Herman M. (2021). Quantitative evaluation of footwear evidence: initial workflow for an end-to-end system. J. Forensic Sci..

[47] Chapman R., Summersby S., Lang T., Raymond J., Ballantyne K. (2023). Novices cannot fill the examiners' shoes: evidence of footwear examiners' expertise in shoe comparisons. Sci. Justice.

[48] Robertson B., Vignaux G.A., Berger C.E.H. (2011). Extending the confusion about bayes. Mod. Law Rev..

[49] Champod C., Evett I.W., Jackson G. (2004). Establishing the most appropriate databases for addressing source level propositions. Sci. Justice.

[50] Hancock S., Morgan-Smith R., Buckleton J. (2012). The interpretation of shoeprint comparison class correspondences. Sci. Justice.

[51] Reel S., Harris R., Reidy S., Chambers J. (2022). The application of TreadMatch scans to aid the process of footwear mark comparison. Sci. Justice.

[52] Biedermann A., Taroni F., Champod C. (2012). How to assign a likelihood ratio in a footwear mark case: an analysis and discussion in the light of R v T, Law. Probability and Risk.

[53] SWGTREAD (2013).

[54] National Footwear Reference Collection (2024).

[55] Bodziak W.J. (2012). Traditional conclusions in footwear examinations versus the use of the bayesian approach and likelihood ratio: a review of a recent UK appellate court decision. Law Probab. Risk.

[56] Bodziak W.J., Hammer L., Johnson G.M., Schenck R. (2012). Determining the significance of outsole wear characteristics during the forensic examination of footwear impression evidence. J. Forensic Ident..

[57] Skerrett J., Neumann C., Mateos-Garcia I. (2011). A Bayesian approach for interpreting shoemark evidence in forensic casework: accounting for wear features. Forensic Sci. Int..

[58] Wiesner S., Shor Y., Tsach T., Kaplan-Damary N., Yekutieli Y. (2020). Dataset of digitized RACs and their rarity Score analysis for strengthening shoeprint evidence. J. Forensic Sci..

[59] Smale A.N., Speir J.A. (2023). Estimate of the random match frequency of acquired characteristics in a forensic footwear database. Sci. Justice.

[60] Stone R.S. (2006). Footwear examinations: mathematical probabilities of theoretical individual characteristics. J. Forensic Ident..

[61] Kaplan D., Mandel M., Wiesner S., Yekutieli Y., Shor Y., Spiegelman C. (2018). Dependence among randomly acquired characteristics on shoeprints and their features. Forensic Sci. Int..

[62] Bily C., Mathias C. (2017). Ethylene vinyl acetate outsoles and acquired characteristics. J. Forensic Ident..

[63] Richetelli N., Bodziak W.J., Speir J.A. (2019). Empirically observed and predicted estimates of chance association: estimating the chance association of randomly acquired characteristics in footwear comparisons. Forensic Sci. Int..

[64] Stoney D. (1992). Reporting of highly individual genetic typing results: a practical approach. J. Forensic Sci..

[65] Koehler J.J., Saks M.J. (2009). Individualization claims in forensic science: still unwarranted. Brook. L. Rev..

[66] Saks M.J., Koehler J.J. (2008). The individualization fallacy in forensic science evidence. Vanderbilt Law Rev..

[67] Wilson H.D. (2012). Comparison of the individual characteristics in the outsoles of thirty-nine pairs of adidas supernova classic shoes. J. Forensic Ident..

[68] Girod A., Champod C., Ribaux O. (2008).

[69] Benedict I., Corke E., Morgan-Smith R., Maynard P., Curran James M., Buckleton J., Roux C. (2014). Geographical variation of shoeprint comparison class correspondences. Sci. Justice.

[70] Facey O.E., Davis R.J. (2011). Re: expressing evaluative opinions; A position statement. Sci. Justice.

[71] Morgan R., Meakin G., French J., Nakhaeizadeh S. (2020).

[72] Morgan R.M., Earwaker H., Nakhaeizadeh S., Harris A.J.L., Rando C., Dror I.E., Laycock G., Tilley N., Wortley R., Sidebottom A. (2019). Routledge Handbook of Crime Science.

[73] Wen Z., Smith R.M., Connor M., Curran J.M. (2024). A ruler detection method for auto-adjusting scales of shoeprint images. Sci. Justice.

[74] Wellman M.P., Henrion M. (1993). Explaining “explaining away,”. IEEE Trans. Pattern Anal. Mach. Intell..

[75] Gittelson S., Berger C.E.H., Jackson G., Evett I.W., Champod C., Robertson B., Curran J.M., Taylor D., Weir B.S., Coble M.D., Buckleton J.S. (2018). A response to “Likelihood ratio as weight of evidence: a closer look” by lund and Iyer. Forensic Sci. Int..

[76] Steele C., Balding D. (2014).

[77] Neumann C., Kaye D., Jackson G., Reyna V., Ranadive A. (2016). Presenting quantitative and qualitative information on forensic science evidence in the courtroom. Chance.

[78] Agena Ltd, https://www.agena.ai/, (n.d.).

[79] (2011). Moss Side House, Court Told.

